# Comparison of commercial and in-house tissue-based and cell-based assays for the detection of autoantibodies targeting neuronal surface proteins: a prospective cohort study

**DOI:** 10.3389/fimmu.2025.1563877

**Published:** 2025-04-15

**Authors:** David Goncalves, Marie Benaiteau, Véronique Rogemond, Sterenn Closs, Anne-Laurie Pinto, Maroua Dhairi, Marine Villard, Géraldine Picard, Nicole Fabien, Jérôme Honnorat

**Affiliations:** ^1^ Service d’immunologie biologique, Hôpital Lyon Sud, Hospices Civils de Lyon, Pierre-Bénite, France; ^2^ French Reference Centre on Paraneoplastic Neurological Syndromes and Autoimmune Encephalitis, Hospices Civils de Lyon, Hôpital Neurologique, Bron, France; ^3^ MeLiS-UCBL-CNRS UMR 5284, INSERM U1314, Université Claude Bernard Lyon 1, Lyon, France

**Keywords:** autoimmune encephalitis, paraneoplastic neurological syndromes, autoantibodies, diagnostic test, immunofluorescence assays, tissue-based assay, cell-based assay

## Abstract

**Introduction:**

The detection of antibodies targeting neuronal antigens is a keystone for the diagnosis of autoimmune encephalitis (AE) and paraneoplastic neurological syndromes (PNS). This study aimed to compare the performance of a commercial tissue-based immunofluorescence assay (cIFA) to that of an inhouse IFA (hIFA) for the screening of autoantibodies targeting neuronal surface proteins in the cerebrospinal fluid (CSF) and to compare the performance of commercial cell-based assays (cCBA) to that of in-house CBA (hCBA) in serum samples.

**Methods:**

Between March and June 2021, 2135 CSF samples and 524 serum samples from 2283 patients referred to the French Reference Center on PNS and AE were prospectively included. CSF samples were all tested using 3 different assays: cIFA, hIFA, and cCBA. Serum samples were all tested using at least 1 cCBA and 1 hCBA for the detection of the following autoantibodies: CASPR2, GABABR, and LGI1.

**Results & Discussion:**

Among the 2135 CSF tested, 93 (4.4%) were positive using both cIFA and hIFA, 1 (0.05%) was positive using only cIFA, and 6 (0.3%) were positive using only hIFA. Among the double-positive samples, 37 (39.8%) were positive using cCBA for the following autoantibodies: anti-NMDAR (n=16), -LGI1 (n=8), -CASPR2 (n=7), -GABABR (n=5), and –DPPX (n=1) autoantibodies. The remaining 56 (60.2%) double-positive samples were negative using cCBA and additional tests were performed to identify the autoantibodies according to the pattern observed on the IFA. The only sample positive using cIFA but negative using hIFA was positive for anti-LGI1 autoantibodies using cCBA. Among the 6 samples negative using cIFA but positive using hIFA, only one sample was positive with cCBA for anti-NMDAR autoantibodies. These data indicate that, in CSF, cIFA and hIFA performed similarly for the detection of autoantibodies targeting neuronal surface proteins.

Regarding serum samples, cCBA and hCBA were both positive in 3 patients for CASPR2, 4 patients for LGI1, and 1 patient for GABABR. A positive cCBA and negative hCBA was observed in 2 patients for LGI1 and 4 patients for GABABR. A lack of specificity of GABABR cCBA is suspected as CSF explorations were negative in 3 of these patients and none presented clinical features highly suggestive of AE.

## 1 Introduction

The detection of antibodies targeting neuronal surface proteins or intracellular antigens is a keystone for the diagnosis and treatment of autoimmune encephalitis (AE) and paraneoplastic neurological syndromes (PNS) (1–3). Over the past several years, commercial kits have been developed and are increasingly used in laboratories to screen for the presence of these autoantibodies (aAbs). A strategy commonly applied consists in a screening assay using a tissue-based immunofluorescence assay (IFA) or immunohistochemistry assay followed by a test to identify the aAbs (cell-based assays [CBA], immunodots…) (4–6). However, tissue-based assays are not always performed as they require expertise and are difficult to read in serum samples due to an intense non-specific staining. In such instances, some laboratories may use only CBA or immunodots to detect and identify the aAbs, even though several studies have reported a high rate of false-positive and false-negative results using these techniques alone (7–10). Moreover, the sensitivity and the specificity of the detection of aAbs targeting neuronal surface proteins also varies according to the sample type tested (serum or cerebrospinal fluid [CSF]) and the aAb detected. For instance, anti-N-methyl-D-aspartate receptor (NMDAR) aAbs are highly specific of anti-NMDAR encephalitis when positive in CSF while an isolated positivity in serum has been described in other diseases and in healthy subjects (11–13). Conversely, serum testing for anti- contactin-associated protein-like 2 (CASPR2), anti- gamma-aminobutyric B receptor (GABABR), and anti- leucine-rich glioma inactivated protein 1 (LGI1) aAbs seems to be more sensitive and specific than CSF testing (13, 14). Assessing the performance of tissue-based and cell-based assays in CSF and serum thus appears essential in order to avoid misdiagnoses and delays in treatment.

This prospective study aimed to compare the performance of a commercial IFA (cIFA) to that of an in-house IFA (hIFA) for the screening of aAbs targeting neuronal surface proteins in the cerebrospinal fluid (CSF) of patients with a suspicion of AE or PNS and to compare the performance of commercial CBA (cCBA) to that of in-house CBA (hCBA) in serum samples.

## 2 Materials and methods

### 2.1 Patients and samples

Between March and June 2021, we prospectively included 2135 CSF and 524 serum samples from 2283 patients that were referred to the French Reference Center on PNS and AE for a screening of aAbs targeting neuronal surface proteins. CSF samples were all tested using 3 different assays: cIFA, hIFA, and cCBA. In case of positivity in at least 1 of the 3 assays, additional tests were performed to confirm and identify the aAb. Serum samples were all tested using at least 1 cCBA and 1 in-house CBA (hCBA) for the detection of at least 1 of the 3 following aAbs, according to the clinician’s request: CASPR2, GABABR, and LGI1. The restriction of serum testing to these 3 aAbs was driven by the lack of specificity of certain aAbs in serum, especially anti-NMDAR aAbs, and the rarity of other aAbs (anti- alpha-amino-3-hydroxyl-5-methyl-4-isoxazole-propionate receptor [AMPAR] or anti- dipeptidyl-peptidase 6 [DPPX] aAbs) (11–13). Based on the screening strategy routinely applied in the reference center, serum samples were not tested using cIFA nor hIFA, due to a more intense non-specific staining that makes reading more difficult compared to CSF. Samples that were referred to the reference center with a request to test only aAbs targeting intracellular antigens were not included in the study.

### 2.2 Commercial tissue-based indirect immunofluorescence assay and commercial cell-based assays

All CSF samples were tested using the Euroimmun cIFA (FA 111a-1010-3, Euroimmun, Lübeck, Germany) on rat cerebellum and hippocampus. All CSF and serum samples were examined using the Autoimmune Encephalitis Mosaic 6 cCBA (FA 112d-1010-6, Euroimmun), which allows for the simultaneous detection of aAbs targeting NMDAR, LGI1, CASPR2, AMPAR, GABABR, and DPPX. Following the manufacturer’s recommendation, CSF was tested undiluted whereas serum samples were incubated at a 1:10 dilution. The cIFA and cCBA stainings were assessed by 2 independent experts (DG and NF).

### 2.3 In-house tissue-based indirect immunofluorescence assay

CSF samples were all tested using hIFA on rat brain sections, as previously described (7). Briefly, rat brains were cut in half and immediately frozen in isopentane at −50°C for 2 minutes. The frozen brains were cut into 12-µm-thick sagittal sections. Brain sections were blocked in phosphate-buffered saline (PBS) containing 3% bovine serum albumin and 3% normal goat serum for 1 hour. Patient CSF was then incubated overnight at room temperature (dilution 1/10). Slides were washed 3 times in PBS and incubated with secondary antibody (goat anti-human coupled to Invitrogen Alexa 488; Thermo Fisher Scientific, Waltham, MA) for 1 hour at room temperature. After 3 washes, slides were mounted in Mowiol medium (Sigma-Aldrich, Saint-Louis, MO). The hIFA stainings were assessed by 2 independent experts (VR and JH).

### 2.4 In-house cell-based assay

Serum samples were all tested using hCBA for the detection of CASPR2, GABABR, and/or LGI1, according to the clinician’s request. CSF samples were tested using hCBA only in case of positivity on the cCBA. Briefly, human embryonic kidney (HEK) 293T cells were cultured on glass coverslips in Dulbecco’s modified Eagle’s medium and were then transiently transfected with cDNAs coding for the recombinant protein of interest. Twenty-four hours after transfection, coverslips were washed, fixed, and incubated with the samples. After 3 PBS washes, a goat anti-human coupled to Invitrogen Alexa 555 (Thermo Fisher Scientific) secondary antibody was incubated.

### 2.5 Additional tests

In case of positivity on cIFA and/or hIFA, additional tests were performed on CSF samples. The choice of additional tests was done according to the pattern observed on the IFA: commercial immunodot (Euroline PNS 12 Ag, DL 1111-1601-7 G, Euroimmun), commercial ELISA for anti-glutamic acid decarboxylase (GAD) antibodies (3802, Medipan, Dahlewitz, Germany), and/or in-house western-blot (for anti-Hu, anti-CV2, and anti-amphiphysin). Commercial assays were performed according to the manufacturer’s recommendations. In-house western-blots were performed as previously described (7). Briefly, HEK293T cells were transfected with a plasmid of interest. Then, cell lysates were subjected to SDS-polyacrylamide gel electrophoresis and then transferred on a membrane. CSF samples were then incubated overnight with the membrane. A peroxidase goat anti-human IgG (Jackson ImmunoResearch, Cambridgeshire, United Kingdom) was used as secondary antibody and chromogenic substrate (Fast 3,3′ Diaminobenzidine tablet, Sigma-Aldrich) was used to show antibody fixation.

### 2.6 Analysis of clinical data & patient consent

In case of discrepant or atypical results, the clinical data obtained from medical records was reviewed by a single neurologist (MB). This led to the classification as either possible, probable, or definite AE or PNS according to the diagnostic criteria for AE and PNS (15, 16), as inconsistent with AE or PNS if an alternative, non-autoimmune diagnosis explaining the symptoms was retained, or as inconclusive in case of insufficient data and/or rapid death. Laboratory analyses were conducted while being blinded to the anonymized clinical data and the clinical analysis was performed while being blinded to the laboratory analysis. The Scientific and Ethical Committee of the Hospices Civils de Lyon approved the study (IFI-NEURO23-5024).

### 2.7 Statistical analysis

Results are expressed as numbers and percentages. The sensitivity of cIFA was determined with 2-way contingency table analysis using Microsoft Excel 2013 (Microsoft Corporation).

## 3 Results

### 3.1 CSF samples

**Figure 1:**
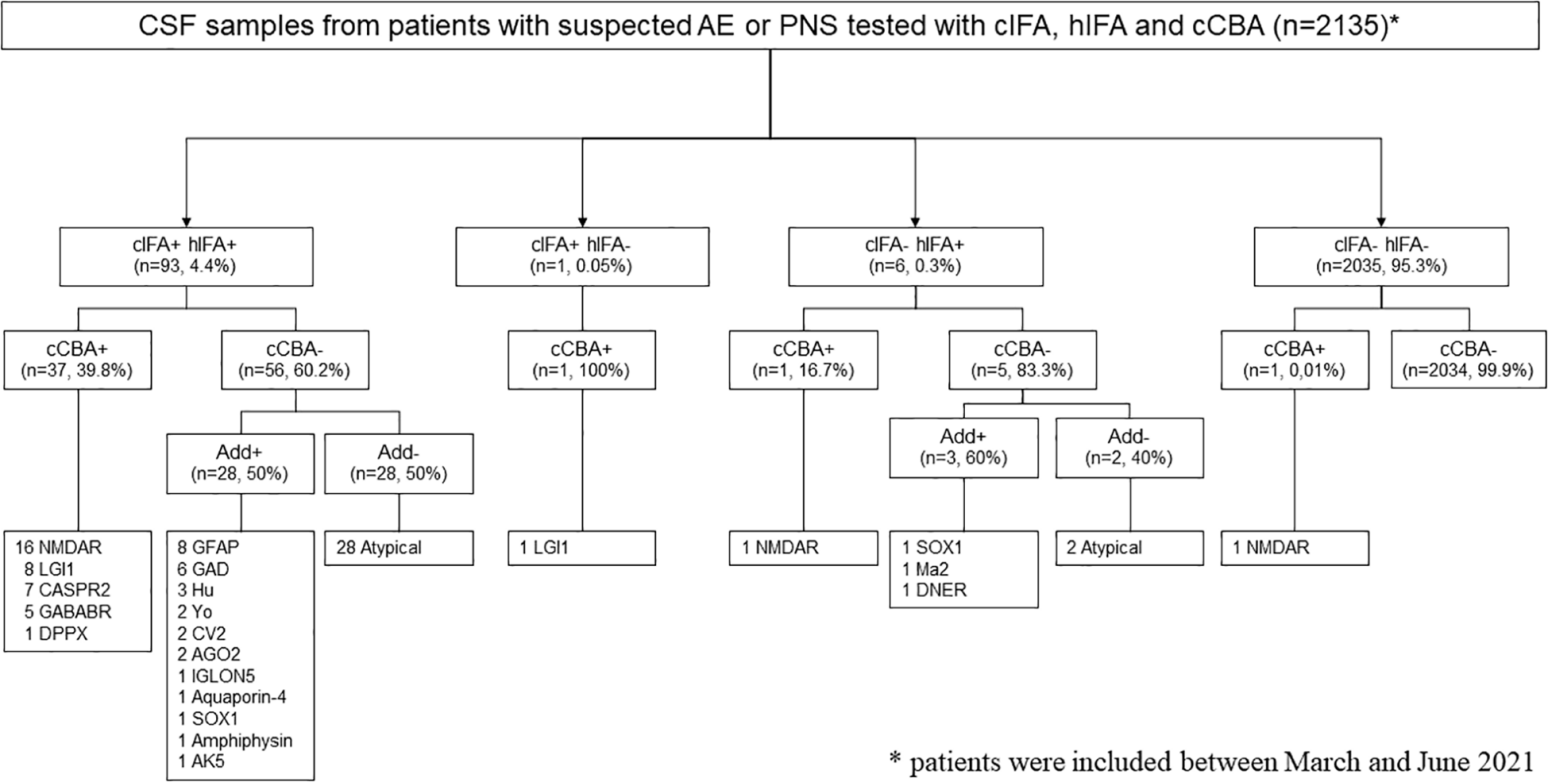
Flow diagram of autoantibody detection in cerebrospinal fluid (CSF) samples of patients with a suspicion of autoimmune encephalitis (AE) or paraneoplastic neurological syndrome (PNS). Add, additional tests; AGO2, argonaute protein 2; AK5, adenylate kinase 5; CASPR2, contactin-associated protein-like 2; cCBA, commercial cell-based assay; cIFA, commercial tissue-based immunofluorescence assay; DNER, Tr/delta/notchlike epidermal growth factor-related receptor; DPPX, dipeptidyl-peptidase 6; GABABR, gamma-aminobutyric B receptor; GAD, glutamic acid decarboxylase; GFAP, glial fibrillary acidic protein; hIFA, in-house tissue-based immunofluorescence assay; IGLON5, Ig-like domain-containing protein 5; LGI1, leucine-rich glioma inactivated protein 1; NMDAR, N-methyl-D-aspartate receptor; SOX1, Sry-like high-mobility group box 1.

Among the 2135 CSF tested, 93 (4.4%) were positive using both cIFA and hIFA, 1 (0.05%) was positive using only cIFA, and 6 (0.3%) were positive using only hIFA (**Figure 1**).

**Figure 2:**
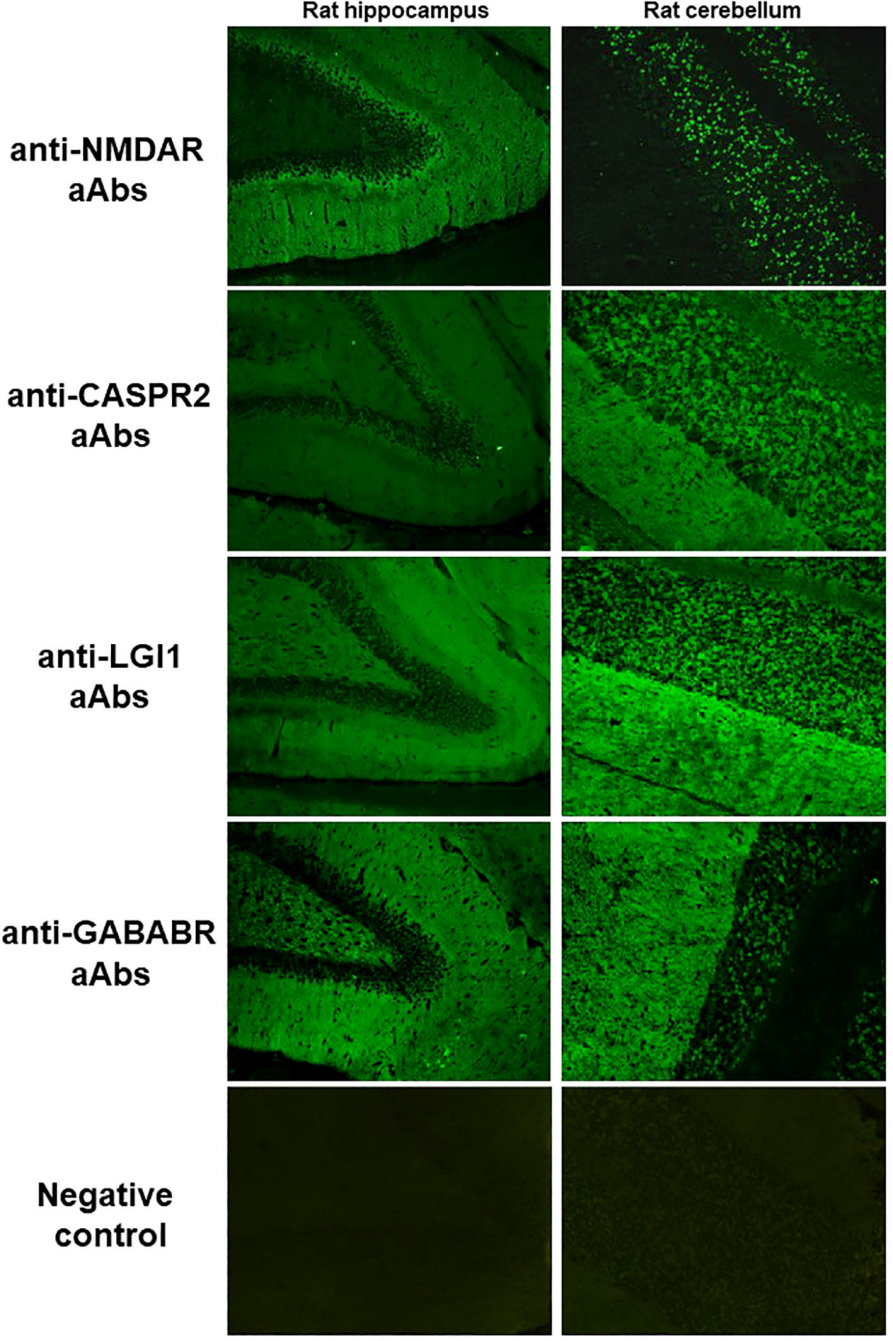
Typical pattern on cIFA of anti-NMDAR, -CASPR2, -LGI1, -GABABR aAbs and a negative control. For anti-NMDAR aAbs, in the hippocampus, the molecular layer of the dentate gyrus is stained following a gradient, with a more intense fluorescence near the dentate granule cell layer; in cerebellum, the granular layer is stained. For anti-CASPR2 aAbs, in the hippocampus, the molecular layer of the dentate gyrus and the dentate hilus are stained homogenously; in the cerebellum, the granular and molecular layers are stained with the same intensity. For anti-LGI1 aAbs, in the hippocampus, the molecular layer of the dentate gyrus and the dentate hilus are stained with a decrease in signal in the inner layer of the molecular layer; in the cerebellum, the molecular layer shows an intense staining while the granular layer staining is mild. For anti-GABABR aAbs, in the hippocampus, the molecular layer of the dentate gyrus and the dentate hilus are stained homogenously; in the cerebellum, the molecular layer shows an intense staining while the granular layer staining is mild. CASPR2, contactin-associated protein-like 2; GABABR, gamma-aminobutyric B receptor; LGI1, leucine-rich glioma inactivated protein 1; NMDAR, N-methyl-D-aspartate receptor.

Among the double-positive samples (cIFA+ hIFA+, **Figure 2**), 37 (39.8%) were positive using cCBA. All 37 samples were then confirmed using hCBA for the following aAbs: anti-NMDAR (n=16), -LGI1 (n=8), -CASPR2 (n=7), -GABABR (n=5) and –DPPX (n=1) aAbs. No anti-AMPAR aAbs were detected.

**Figure 3:**
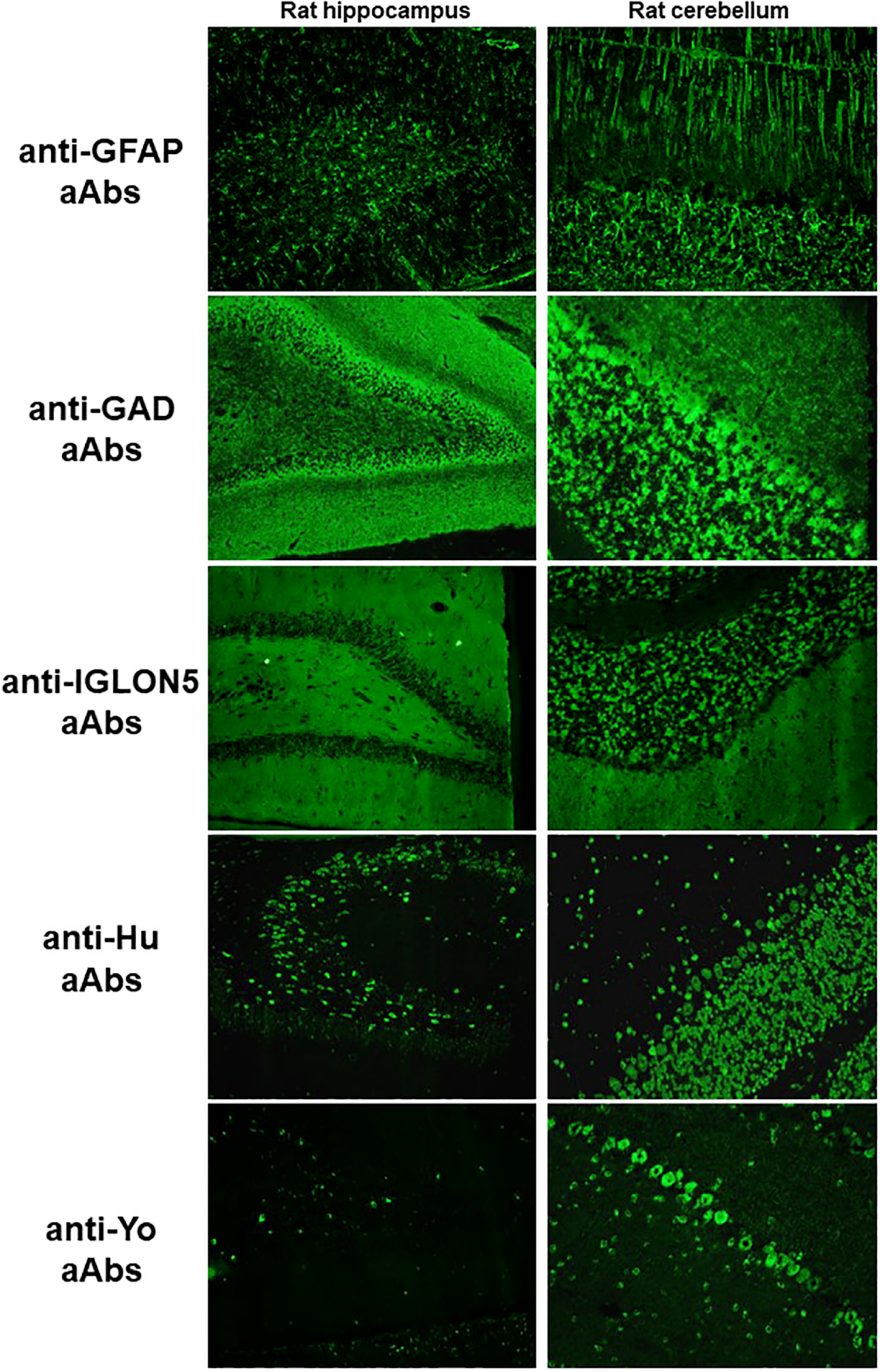
Typical pattern on cIFA of anti-GFAP, -GAD, -IGLON5, -Hu, and -Yo aAbs. For anti-GFAP aAbs, in the hippocampus, there is a filamentous staining associated with an astrocyte staining in the dentate hilus; in the cerebellum, the radial glia of Bergmann in the molecular layer and astrocytes in the granular layer are stained. For anti-GAD aAbs, in the hippocampus, there is a strong staining of all the layers due to the cytoplasmic expression of GAD; in the cerebellum, the molecular layer shows a granular staining with dots while the granular layer and Purkinje cells show an intense cytoplasmic staining. For anti-IGLON5 aAbs, in the hippocampus, the molecular layer of the dentate gyrus and the dentate hilus are stained homogenously; in the cerebellum, the granular and molecular layers are stained with the same intensity. For anti-Hu aAbs, in the hippocampus, the nuclei of the dentate granule cells are stained while the cytoplasm and the nuclei of cells in the stratum pyramidale are stained; in the cerebellum, the nuclei of cells in all layers are stained as well as Purkinje cell cytoplasms. For anti-Yo aAbs, in the hippocampus, the cytoplasms of some cells in the hilus are stained; in the cerebellum, Purkinje cell cytoplasms are stained. GAD, glutamic acid decarboxylase; GFAP, glial fibrillary acidic protein; IGLON5, Ig-like domain-containing protein 5.

The remaining 56 (60.2%) double-positive samples were negative using cCBA and additional tests were performed to identify the aAb according to the pattern observed on the IFA (**Figure 3**). The aAb were identified in 28 (50%) of these samples targeting the following antigens: glial fibrillary acidic protein (GFAP, n=8), GAD (n=6), Hu (n=3), Yo (n=2), CV2/collapsing response-mediator protein 5 (CRMP5, n=2), argonaute protein 2 (AGO2, n=2), Ig-like domain-containing protein 5 (IGLON5, n=1), aquaporin-4 (n=1), Sry-like high-mobility group box 1 (SOX1, n=1), amphiphysin (n=1), and adenylate kinase 5 (AK5, n=1). In the remaining 28 (50%) samples, no aAbs were identified; these samples were therefore classified as atypical. To explore the clinical significance of these atypical stainings, we reviewed the clinical data when available (25 patients, **Table 1**): 14 (56%) patients met the diagnostic criteria for possible or probable AE or PNS, 1 (4%) met the diagnostic criteria for definite PNS after an *a posteriori* identification of anti-RGS8 aAb, and 1 (4%) patient presented with an ICI-related encephalitis. An alternative, non-autoimmune diagnosis was retained for 4 patients (16%) and for the remaining 5 (20%), no diagnosis was reached due to atypical symptoms, insufficient explorations, or rapid death.

**Table 1:** Clinical characteristics of patients with atypical cerebrospinal fluid staining.

Patient	Sex	Age	Cancer	Symptoms	Paraclinical examinations	Outcome	Findings: AE and PNS definition criteria (15, 16)
1	M	50	Discovery of Hodgkin Lymphoma	Rapid progressive cerebellar syndrome	Normal MRI, inflammatory CSF (pleocytosis and presence of OCB). Posteriori identification of anti-RGS8	Overall stability after hematologic chemotherapy, IV immunoglobulins, and rituximab	Definite PNS
2	M	59	Discovery of a Merkel Carcinoma	Cerebellar syndrome and Lambert-Eaton myasthenic syndrome	Normal MRI, inflammatory CSF (pleocytosis and presence of OCB)	Improvement after IV immunoglobulins, corticosteroids, cyclophosphamide, complete resection of the carcinoma, and radiotherapy	Probable PNS
3	F	74	NA	Psychiatric disorder followed by epilepsy, cognitive and impaired consciousness or alertness, dysautonomia and movement disorders***.	Abnormal MRI, inflammatory CSF (pleocytosis and presence of OCB)	Good progressive recovery after corticosteroids and IV immunoglobulins	Probable AE
4	M	77	Discovery of a neuroendocrine pulmonary cancer	Severe motor and sensitive neuropathy	NA	Rapid death	Probable PNS
5	M	79	Discovery of a neuroendocrine pulmonary cancer	Rapid progressive cerebellar syndrome	Normal MRI, inflammatory CSF (pleocytosis)	Rapid death	Probable PNS
6	M	18	Negative assessment	Severe limbic encephalitis followed by cerebellar syndrome	Bitemporal and cerebellar T2 hypersignals on MRI, inflammatory CSF (pleocytosis and presence of OCB)	Slow improvement after corticosteroids and mitoxantrone but severe handicap	Probable AE
7	M	63	Discovery of an undifferentiated carcinoma on adenopathy, unknown primary site	Rapid progressive cerebellar syndrome and dysautonomia	Normal MRI, inflammatory CSF (presence of OCB)	NA	Possible or probable PNS
8	F	50	Discovery of a breast carcinoma	Acute limbic encephalitis	Abnormal MRI, inflammatory CSF (pleocytosis and presence of OCB)	Relapse in 2024	Probable PNS
9	M	68	Discovery of a metastatic neuroendocrine pulmonary cancer	Rapid progressive cerebellar syndrome and severe retinopathy	Normal MRI, inflammatory CSF (presence of OCB)	Death one year later	Probable PNS
10	M	72	Pulmonary adenocarcinoma treated by ICI	Impaired consciousness or alertness and cognitive disorders	Normal MRI, normal CSF	Complete resolution with corticosteroids	ICI-related encephalitis
11	M	85	Negative assessment	Subacute neuronopathy, rapid progressive cerebellar syndrome and hyponatremia	Normal cerebral and medullar MRI	Bedridden patient	Possible PNS
12	F	69	Negative assessment	Rapid progressive cerebellar syndrome	Normal MRI, inflammatory CSF (presence of OCB).Positivity of anti-Ago2 and anti-SSA	No improvement with several immunotherapies	Possible PNS or AE
13	F	50	NA	General alteration*, opsoclonus-myoclonus, rapid progressive cerebellar syndrome, cognitive disorders	Inflammatory CSF (presence of OCB)	NA	Possible PNS
14	M	31	NA	Meningoencephalitis, optic neuritis, cerebellar syndrome	White matter T2 hypersignals on MRI, inflammatory CSF (pleocytosis)	Improvement after corticosteroids and plasmapheresis	Possible AE
15	M	63	Negative assessment	Sensitive and small fiber neuropathy in context of dysimmunity	Inflammatory CSF (presence of OCB)	Improvement after Iv immunoglobulins	Possible dysimmune neuropathy
16	M	69	Metastatic prostatic carcinoma	Acute dysarthria, swallowing disorders**, and movements disorders***	Normal MRI, inflammatory CSF (pleocytosis) without malignant cell	Rapid death (inhalation pneumonitis)	Possible PNS
17	F	75	Metastatic bilateral breast carcinoma	Subacute cognitive disorders, cerebellar syndrome, vomiting, hyponatremia (115 mmol/l)	Normal MRI, normal CSF	Rapid death	Inconclusive findings between probable PNS and carcinomatous meningitis
18	F	72	NA	HSV1 meningoencephalitis without other distinguishable symptoms of AE	Extension of Flair/T2 hypersignals in the left temporo-insular sequelae, persistence of inflammatory CSF (gradually diminished pleocytosis after antiviral treatment.	Slow improvement without immunotherapy	Inconclusive findings between herpetic encephalitis and autoimmune encephalitis post-herpetic encephalitis
19	F	49	Malignant thymoma with incomplete resection	Autoimmune myasthenia and progressively worsening amyotrophic motor deficit leading to suspicion of motor neuron disease	ENMG: normal sensory and motor conduction, active and chronic denervation patterns in the 4 limbs and bulbar region, normal CSF, normal brain and medullar MRI	No improvement after immunoglobulins	Inconclusive findings
20	H	69	Negative assessment	Fluctuating paraparesis and cognitive impairment	Normal brain and medullar MRI, normal CSF, normal ENMG	NA	Inconclusive findings
21	H	78	NA	Extrapyramidal and dysexecutive syndromes, behavioral symptoms for the past year	Severe leukopathy on MRI, inflammatory CSF (pleocytosis)	Patient refusal of follow-up and explorations	Inconclusive findings
22	F	78	Chronic lymphocytic leukemia	Walking difficulties and cognitive slowing	Subcortical T2 hypersignals on MRI. Neuroborreliosis and meningeal location of lymphocytic leukemia on CSF	NA	Alternative diagnosis: Neuroborreliosis and meningeal location of lymphocytic leukemia
23	H	75	NA	Epileptic seizures after COVID-19 vaccine	Normal MRI, normal CSF, normal EEG	Complete recovery and patient refusal of follow-up	Alternative diagnosis: symptomatic seizures without encephalitis
24	H	70	Suspicion of metastatic pulmonary cancer	Cerebellar syndrome followed by cognitive disorders and agitation	Suspicion of brain metastases on MRI,	Rapid death, before biopsy	Alternative diagnosis: probable brain metastases
25	H	75	NA	Left L3 radiculopathy with motor deficit on excluded hernia	Non-inflammatory CSF	NA	Alternative diagnosis: compressive radiculopathy

AE, autoimmune encephalitis; CSF, cerebrospinal fluid; EEG, electroencephalogram; ENMG, electroneuromyography; F, female; HSV1, herpes simplex virus type 1; ICI, immune checkpoint inhibitor; IV, intravenous; M, male; MRI, magnetic resonance imaging; NA, not available; OCB, oligoclonal bands; PNS, paraneoplastic neurological syndrome; RGS8, regulator of G-protein signaling 8.

*General alteration = state associating intense asthenia, anorexia, and weight loss.

**Swallowing disorders = cerebellar or brainstem syndrome.

***Movement disorders = tremor, dystonia, choreic movements, myoclonus, and dyskinesia.

The only sample positive using cIFA but negative using hIFA was positive using LGI1-cCBA but negative using LGI1-hCBA. This sample was drawn from a 60-year-old man with a typical clinical presentation of anti-LGI1 aAbs encephalitis associating temporal seizures, confusion with spatial temporal disorientation, and left temporal Flair hyperintensities on magnetic resonance imaging (MRI).

Among the 6 samples negative using cIFA but positive using hIFA, only one sample was positive with cCBA for anti-NMDAR aAbs, confirmed with NMDAR-hCBA. This sample was drawn from a 31-year-old man presenting seizures, memory impairment, and psychobehavioral changes consistent with anti-NMDAR aAbs encephalitis. The remaining 5 samples were negative using cCBA and additional tests were performed to identify the aAb according to the pattern observed on the hIFA. The following aAbs were identified in 3 of these samples: SOX1 (n=1), Ma2 (n=1), and Tr/delta/notchlike epidermal growth factor-related receptor (DNER, n=1); the remaining 2 samples were classified as atypical since the aAbs were not identified.

The large majority of the CSF samples were found to be negative using both cIFA and hIFA (2035/2135, 95.2%). In these samples, only one was found to be positive using cCBA for anti-NMDAR aAbs but negative using NMDAR-hCBA. The clinical presentation was not suggestive of an anti-NMDAR aAbs encephalitis since a diagnosis of secondary progressive multiple sclerosis was made based on the clinical and MRI presentation in a 45-year-old man with progressive cognitive disorders.

For the most common neuronal surface proteins identified using cCBA (NMDAR, LGI1, CASPR2, and GABABR), the sensitivity of cIFA and hIFA was evaluated **Table 2**. Overall, sensitivity was high for both cIFA and hIFA (97.4% for both); hIFA appeared to be more sensitive than cIFA for anti-NMDAR aAbs (100% vs. 94.1%, respectively) while cIFA seemed more sensitive than hIFA for anti-LGI1 aAbs (100% vs. 88.9%, respectively).

**Table 2:** Sensitivity of cIFA and hIFA for the detection of aAbs targeting the most common surface antigens (NMDAR, LGI1, CASPR2, GABABR) in CSF.

	Sensitivity [95%CI]	
	cIFA	hIFA
Overall (NMDAR, LGI1, CASPR2, GABABR included)	97.4 % [92.3 – 100]	97.4 % [92.3 – 100]
Anti-NMDAR aAbs	94.1 % [82.9 – 100]	100 %
Anti-LGI1 aAbs	100 %	88.9 % [68.4-100]
Anti-CASPR2 aAbs	100 %	100 %
Anti-GABABR aAbs	100 %	100 %

aAbs, autoantibodies; CASPR2, contactin-associated protein-like 2; CI, confidence interval; cIFA, commercial tissue-based immunofluorescence assay; CSF, cerebrospinal fluid; GABABR, gamma-aminobutyric B receptor; hIFA, in-house tissue-based immunofluorescence assay; LGI1, leucine-rich glioma inactivated protein 1; NMDAR, N-methyl-D-aspartate receptor.

### 3.2 Serum samples

**Figure 4:**
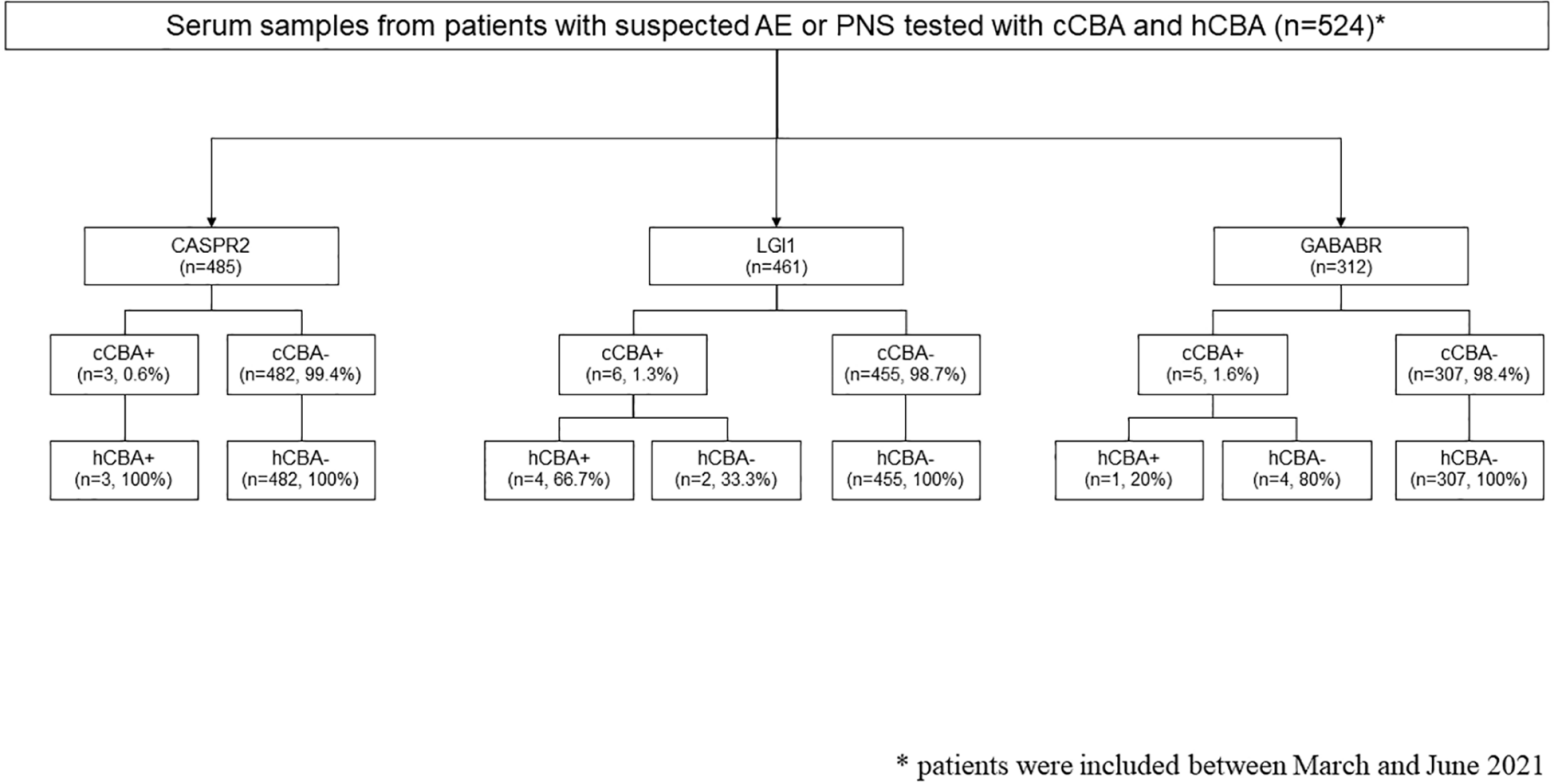
Flow diagram of autoantibody detection in serum samples of patients with a suspicion of autoimmune encephalitis (AE) or paraneoplastic neurological syndrome (PNS). CASPR2, contactin-associated protein-like 2; cCBA, commercial cell-based assay; GABABR, gamma-aminobutyric B receptor; hCBA, in-house cell-based assay; LGI1, leucine-rich glioma inactivated protein 1.

A total of 485 sera were tested for CASPR2 using the cCBA; 3 (0.6%) samples were found to be positive, all confirmed by the hCBA (**Figure 4**). In these patients, 2 (67%) were also positive for anti-CASPR2 aAbs in CSF while 1 (33%) was negative.

Among the 461 sera tested for LGI1, 6 (1.3%) were positive using the cCBA; 4 (66.7%) were confirmed by the hCBA while 2 (33.3%) were negative. In 3 patients with positive cCBA and positive hCBA (75%), anti-LGI1 aAbs were also positive in the CSF while no CSF was tested in 1 patient (25%). One of the discrepant samples (positive cCBA and negative hCBA) was drawn from a 77-year-old man with a typical limbic encephalitis compatible with anti-LGI1 aAbs encephalitis (confusion, seizures, and Flair hyperintensity of medial temporal lobes on MRI). Of note, no CSF testing was ordered for this patient. The other discrepant sample was from a 73-year-old man, who was also positive for anti-GABABR aAbs on cCBA. This patient had a negative CSF analysis and presented with paroxysmal dystonic episodes related to a large thrombosed basilar aneurysm with mass effect on the brainstem, without any other neurological sign consistent with anti-LGI1 or anti GABABR aAbs encephalitis after extensive investigations.

Regarding GABABR, 312 sera were tested and 5 (1.6%) were positive using the cCBA. Only one (20%) was also positive using hCBA. Sera with positive cCBA but negative hCBA (4/5, 80%) were drawn from patients with clinical presentations that were not consistent with anti-GABABR aAbs encephalitis (Gayet-Wernicke-Korsakoff syndrome, infectious encephalopathy, chronic cerebellar ataxia with axonal polyneuropathy suggesting a genetic origin, and the patient described above). The CSF from 3 of these patients were also tested and were negative.

Importantly, no negative cCBA was found to be positive using hCBA.

## 4 Discussion

Based on a large prospective cohort of patients with a suspicion of AE or PNS, the present study found that cIFA enables the detection of aAbs targeting neuronal surface proteins in CSF samples as effectively as hIFA, with some limitations.

Contrary to a recent report describing a high rate of false-negative results using cIFA (34%), particularly in the case of anti-NMDAR aAbs (17), only a small proportion of samples herein were considered as false-negatives using cIFA. Among these, 4 were identified as positive for anti-NMDAR, anti-SOX1, anti-Ma2, and anti-DNER aAbs using hIFA and additional tests. Despite the lower rate of false negative results observed herein, the combination of both tissue-based and cell-based assays may be the best strategy for the detection and identification of certain aAbs, notably anti-NMDAR aAbs. In line with this proposed strategy, a recent report based on a nationwide retrospective cohort shows that combining IFA and CBA increases the sensitivity and specificity for most of the aAbs targeting neuronal surface proteins compared to CBA alone (13). Combining these assays may therefore also limit the risk of false positive results, even though we only observed one case of false positive result herein, with anti-NMDAR aAbs being positive only with cCBA. In this patient, hIFA and hCBA were both negative and the clinical presentation was not suggestive of an anti-NMDAR aAbs encephalitis, highlighting a possible lack of specificity of cCBA when performed alone. Overall, these findings underscore the necessity to combine different assays, such as cIFA and cCBA, to confirm the positivity or negativity of aAbs targeting neuronal surface proteins, especially anti-NMDAR aAbs, since a false-positive result may lead to unnecessary treatment while a false-negative result may lead to a delay in diagnosis and treatment (18, 19).

Although there were only a small number of positive samples using cIFA, hIFA, and cCBA, the present results suggest a similar sensitivity between cIFA and hIFA for the detection of the most common aAbs targeting neuronal surface proteins (NMDAR, LGI1, CASPR2, and GABABR). Further studies are needed to confirm these results as well as to evaluate the sensitivity of these assays for other aAbs targeting neuronal surface proteins or intracellular antigens. Of note, the performance of cIFA, which is a subjective test requiring expertise, seems to be highly dependent on the operator, as illustrated by the high variability observed between laboratories (17, 20). Therefore, in case of negative cIFA and when the clinical suspicion of AE or PNS is high, it still seems necessary to perform additional tests and/or refer the request to reference laboratories, as recommended by the PNS diagnostic criteria (16).

One of the main interests of using cIFA for CSF samples, as illustrated by the present results, is its capacity to detect, using a single test, a large panel of anti-neuronal aAbs otherwise not detected by the cCBA used herein. Even though the samples included in the present study were referred for a screening of aAbs targeting neuronal surface proteins, both the cIFA and hIFA identified some aAbs targeting intracellular antigens; such identification usually requires a different processing of brain tissue. Using a single test for both neuronal surface and intracellular antigens would be clinically relevant but requires to be further evaluated in dedicated studies. From a clinical standpoint, the separation between aAbs targeting neuronal surface proteins or intracellular antigens is tricky since clinical presentation might be similar, especially in the early stages of the disease. The present findings also underline that some samples show an atypical staining on cIFA and hIFA. The review of the clinical records of more than half of these patients enabled the classification into possible or probable AE or PNS, suggesting that some aAbs might be present in the CSF of these patients but that they are targeting a still unknown antigen. However, an atypical staining on cIFA and hIFA was also observed in some patients with a diagnosis other than AE or PNS, indicating a possible lack of specificity of tissue-based assays. The positivity of tissue-based assays in such instances could be due to an immune response secondary to brain damage from other diseases, such as infectious or neurodegenerative diseases.

Anti-neuronal surface protein aAbs are sometimes only positive in the serum, especially anti-CASPR2 and anti-LGI1 aAbs (14). The present findings suggest that cCBA can detect aAbs targeting CASPR2, LGI1, and GABABR as effectively as hCBA since no isolated positivity with hCBA was observed. However, some discrepant results were observed, especially for anti-GABABR aAbs, which were positive only on cCBA for 4 patients with unrelated clinical features and negative explorations in CSF. Previous studies report a lack of sensitivity and specificity for cCBA when performed alone (8, 13, 21) and recommend to combine assays in serum, similarly to what was performed herein in CSF samples.

The rate of positivity in the present cohort was relatively low (<5%). This may be due to the design of the study, as all samples were tested prospectively based only on the clinicians’ request, without controlling for its relevance. Some tests may have thus been requested by non-expert clinicians, as part of their screening work-up, even though the probability for AE or PNS was relatively low. Importantly, the present cohort appears to be representative of patients with suspected AE, as anti-NMDAR followed by anti-LGI1 were the most frequently observed aAbs, and some aAbs were rarely or never observed (i.e. anti-AMPAR aAbs) (22, 23). Finally, since the study was based on the screening strategy of the reference center, in which sample testing can still be performed if only one sample type is sent, paired CSF and serum samples were not available for all patients. We therefore cannot conclude on which sample type should be tested according to the aAb. However, the findings herein suggest that using different assays and/or different sample types may help identify false-positive results, as evidenced by the GABABR results in serum.

In conclusion, cIFA showed a similar performance to that of hIFA for the detection of aAbs targeting neuronal surface proteins in the CSF of patients with a suspicion of AE or PNS. Combining cIFA and cCBA may enable to avoid false-negative and false-positive results. In serum, cCBA appears to perform similarly to hCBA but needs to be further evaluated to assess its specificity, particularly regarding anti-GABABR aAbs.

## Data Availability

The raw data supporting the conclusions of this article will be made available by the authors, without undue reservation.
